# Supplementary material for: A fault-intrusion-tolerant system and deadline-aware algorithm for scheduling scientific workflow in the cloud
Source: PeerJ Comput Sci. 2021 Nov 2;7:e747. doi: 10.7717/peerj-cs.747 (PMC8576554; doi:10.7717/peerj-cs.747)
Supplement: Supplemental Information 6 [file peerj-cs-07-747-s006.zip › Myproject1.1/docs/org/cloudbus/cloudsim/lists/PeList.html]

PeList


JavaScript is disabled on your browser.


- Overview
- Package
- Class
- Use
- Tree
- Deprecated
- Index
- Help

- Prev Class
- Next Class

- Frames
- No Frames

- All Classes

- Summary:
- Nested |
- Field |
- Constr |
- Method

- Detail:
- Field |
- Constr |
- Method


org.cloudbus.cloudsim.lists

## Class PeList

- java.lang.Object
- - org.cloudbus.cloudsim.lists.PeList

- ---

    

  ```
  public class PeList
  extends java.lang.Object
  ```

  PeList is a collection of operations on lists of PEs.

  Since:
  :   CloudSim Toolkit 2.0

- - ### Constructor Summary

    Constructors

    Constructor and Description

    `PeList()`
  - ### Method Summary

    Methods

    | Modifier and Type | Method and Description |
    | --- | --- |
    | `static <T extends Pe>  Pe` | `getById(java.util.List<T> peList, int id)` Gets MIPS Rating for a specified Pe ID. |
    | `static <T extends Pe>  Pe` | `getFreePe(java.util.List<T> peList)` Gets a Pe ID which is FREE. |
    | `static <T extends Pe>  double` | `getMaxUtilization(java.util.List<T> peList)` Gets the max utilization among by all PEs. |
    | `static <T extends Pe>  double` | `getMaxUtilizationAmongVmsPes(java.util.List<T> peList, Vm vm)` Gets the max utilization among by all PEs allocated to the VM. |
    | `static <T extends Pe>  int` | `getMips(java.util.List<T> peList, int id)` Gets MIPS Rating for a specified Pe ID. |
    | `static <T extends Pe>  int` | `getNumberOfBusyPes(java.util.List<T> peList)` Gets the number of BUSY Pe. |
    | `static <T extends Pe>  int` | `getNumberOfFreePes(java.util.List<T> peList)` Gets the number of FREE or non-busy Pe. |
    | `static <T extends Pe>  int` | `getTotalMips(java.util.List<T> peList)` Gets total MIPS Rating for all PEs. |
    | `static <T extends Pe>  boolean` | `setPeStatus(java.util.List<T> peList, int id, int status)` Sets the Pe status. |
    | `static <T extends Pe>  void` | `setStatusFailed(java.util.List<T> peList, boolean failed)` Sets the status of PEs of this machine to FAILED. |
    | `static <T extends Pe>  void` | `setStatusFailed(java.util.List<T> peList, java.lang.String resName, int hostId, boolean failed)` Sets the status of PEs of this machine to FAILED. |

    - ### Methods inherited from class java.lang.Object

      `clone, equals, finalize, getClass, hashCode, notify, notifyAll, toString, wait, wait, wait`

- - ### Constructor Detail


    - #### PeList

      ```
      public PeList()
      ```
  - ### Method Detail


    - #### getById

      ```
      public static <T extends Pe> Pe getById(java.util.List<T> peList,
                              int id)
      ```

      Gets MIPS Rating for a specified Pe ID.

      Parameters:
      :   `id` - the Pe ID
      :   `peList` - the pe list

      Returns:
      :   the MIPS rating if exists, otherwise returns -1


    - #### getMips

      ```
      public static <T extends Pe> int getMips(java.util.List<T> peList,
                               int id)
      ```

      Gets MIPS Rating for a specified Pe ID.

      Parameters:
      :   `id` - the Pe ID
      :   `peList` - the pe list

      Returns:
      :   the MIPS rating if exists, otherwise returns -1


    - #### getTotalMips

      ```
      public static <T extends Pe> int getTotalMips(java.util.List<T> peList)
      ```

      Gets total MIPS Rating for all PEs.

      Parameters:
      :   `peList` - the pe list

      Returns:
      :   the total MIPS Rating


    - #### getMaxUtilization

      ```
      public static <T extends Pe> double getMaxUtilization(java.util.List<T> peList)
      ```

      Gets the max utilization among by all PEs.

      Parameters:
      :   `peList` - the pe list

      Returns:
      :   the utilization


    - #### getMaxUtilizationAmongVmsPes

      ```
      public static <T extends Pe> double getMaxUtilizationAmongVmsPes(java.util.List<T> peList,
                                                       Vm vm)
      ```

      Gets the max utilization among by all PEs allocated to the VM.

      Parameters:
      :   `vm` - the vm
      :   `peList` - the pe list

      Returns:
      :   the utilization


    - #### getFreePe

      ```
      public static <T extends Pe> Pe getFreePe(java.util.List<T> peList)
      ```

      Gets a Pe ID which is FREE.

      Parameters:
      :   `peList` - the pe list

      Returns:
      :   a Pe ID if it is FREE, otherwise returns -1


    - #### getNumberOfFreePes

      ```
      public static <T extends Pe> int getNumberOfFreePes(java.util.List<T> peList)
      ```

      Gets the number of FREE or non-busy Pe.

      Parameters:
      :   `peList` - the pe list

      Returns:
      :   number of Pe


    - #### setPeStatus

      ```
      public static <T extends Pe> boolean setPeStatus(java.util.List<T> peList,
                                       int id,
                                       int status)
      ```

      Sets the Pe status.

      Parameters:
      :   `status` - Pe status, either Pe.FREE or Pe.BUSY
      :   `id` - the id
      :   `peList` - the pe list

      Returns:
      :   true if the Pe status has been changed, false otherwise (Pe id might
          not be exist)


    - #### getNumberOfBusyPes

      ```
      public static <T extends Pe> int getNumberOfBusyPes(java.util.List<T> peList)
      ```

      Gets the number of BUSY Pe.

      Parameters:
      :   `peList` - the pe list

      Returns:
      :   number of Pe


    - #### setStatusFailed

      ```
      public static <T extends Pe> void setStatusFailed(java.util.List<T> peList,
                                        java.lang.String resName,
                                        int hostId,
                                        boolean failed)
      ```

      Sets the status of PEs of this machine to FAILED. NOTE: resName and
      machineID are used for debugging purposes, which is **ON** by default. Use
      `#setStatusFailed(boolean)` if you do not want this information.

      Parameters:
      :   `resName` - the name of the resource
      :   `hostId` - the id of this machine
      :   `failed` - the new value for the "failed" parameter


    - #### setStatusFailed

      ```
      public static <T extends Pe> void setStatusFailed(java.util.List<T> peList,
                                        boolean failed)
      ```

      Sets the status of PEs of this machine to FAILED.

      Parameters:
      :   `failed` - the new value for the "failed" parameter
      :   `peList` - the pe list


- Overview
- Package
- Class
- Use
- Tree
- Deprecated
- Index
- Help

- Prev Class
- Next Class

- Frames
- No Frames

- All Classes

- Summary:
- Nested |
- Field |
- Constr |
- Method

- Detail:
- Field |
- Constr |
- Method
